# Supplementary material for: Selective semihydrogenation of acetylene in ethylene using defect-rich boron nitride catalyst from flux reconstruction
Source: Nat Commun. 2025 Nov 12;16:9948. doi: 10.1038/s41467-025-64886-x (PMC12612240; doi:10.1038/s41467-025-64886-x)
Supplement: Supplementary file 1 — Supplementary Information [file 41467_2025_64886_MOESM1_ESM.pdf]

Supplementary Figures

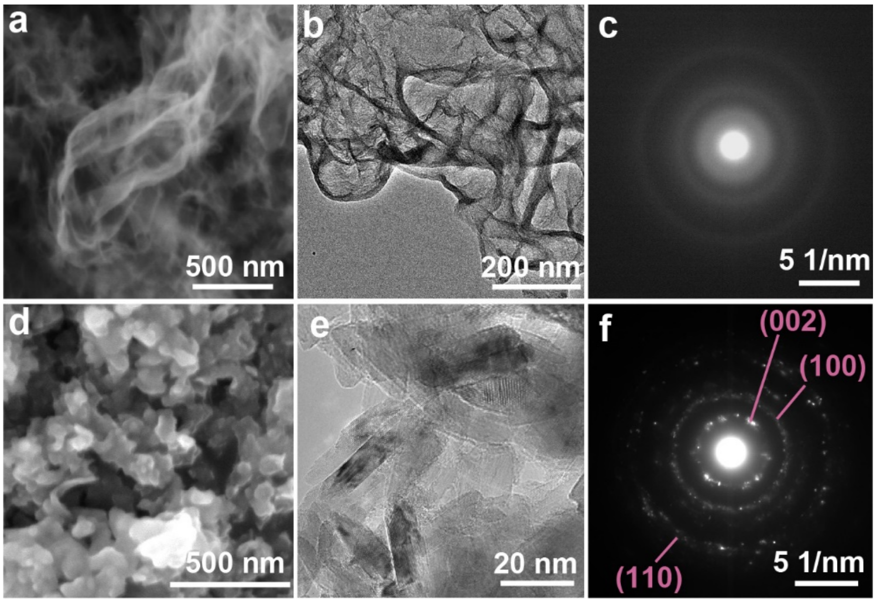

Supplementary Fig. 1. SEM images of AMBN (a) and BN-700 (d); TEM images of AMBN (b) and BN-700 (e); SAED pattern of AMBN (c) and BN-700 (f).

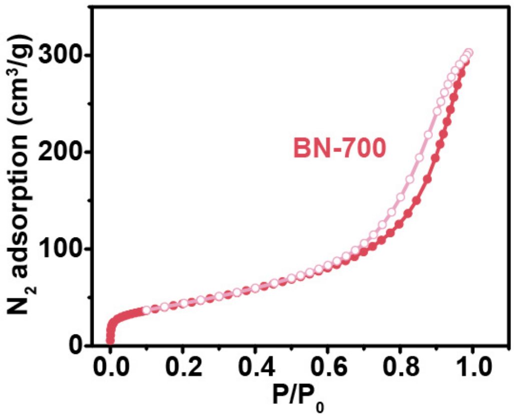

Supplementary Fig. 2. N_2 adsorption-desorption isotherms of BN-700 at 77 K. Source data are provided as a Source Data file.

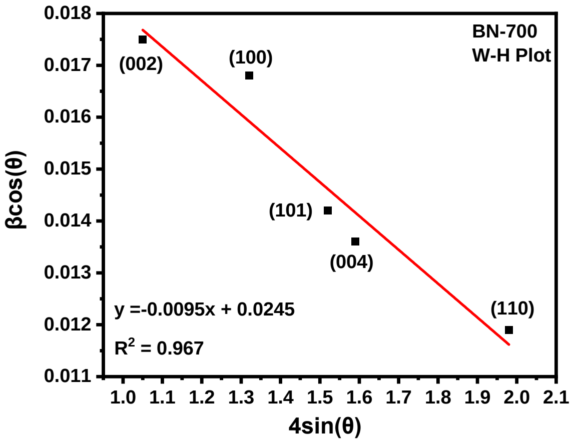

Supplementary Fig. 3. Williamson-Hall plot of BN-700 after instrumental broadening correction. Source data are

provided as a Source Data file.

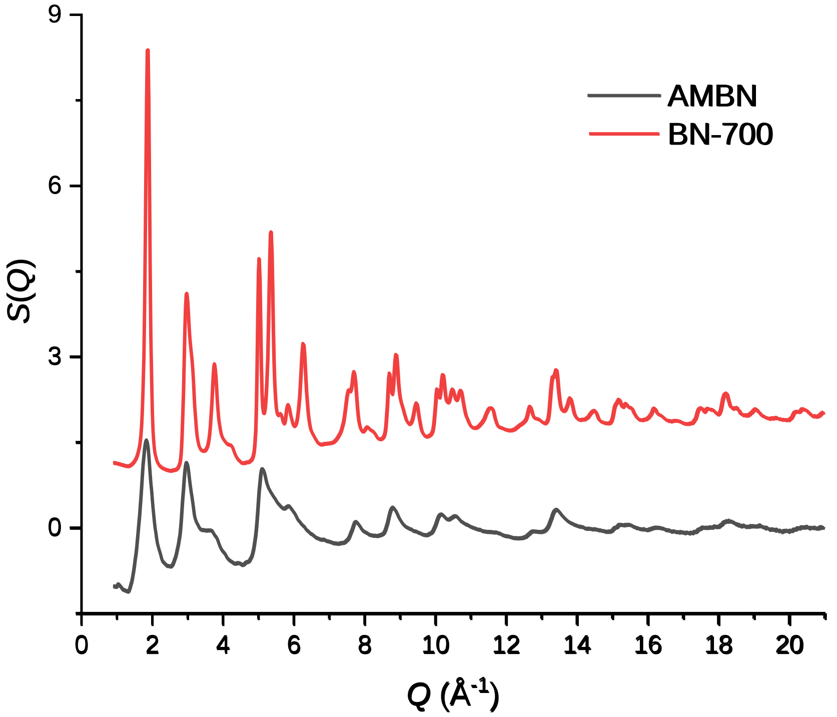

Supplementary Fig. 4. Structure functions, *S(Q)*'s, for the AMBN and BN-700 materials. The data are offset for clarity. Source data are provided as a Source Data file.

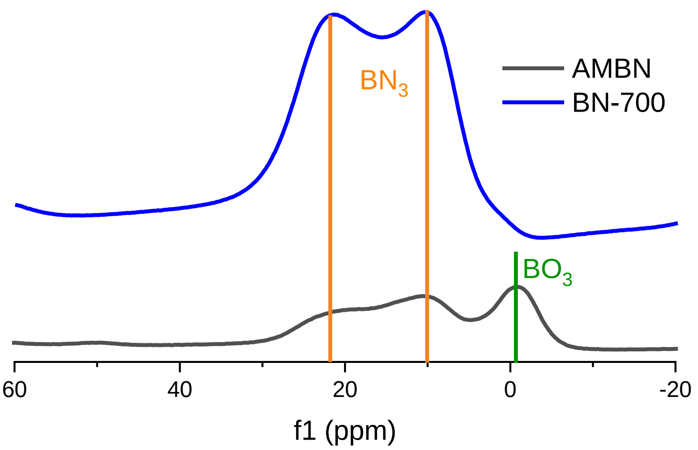

Supplementary Fig. 5. The MAS SS ^{11}B NMR of AMBN and BN-700. Source data are provided as a Source Data file.

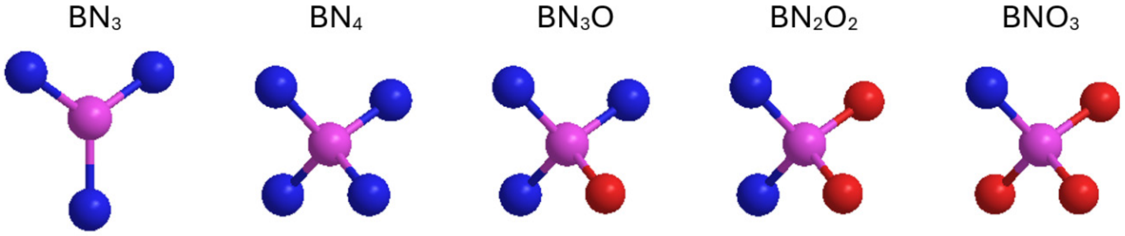

Supplementary Fig. 6. Illustration of tricoordinate BN_3 bonds and tetracoordinated BN_xO_{4-x} (x = 0-3) species. Oxygen, boron, and nitrogen atoms are represented by red, pink, and blue spheres, respectively.

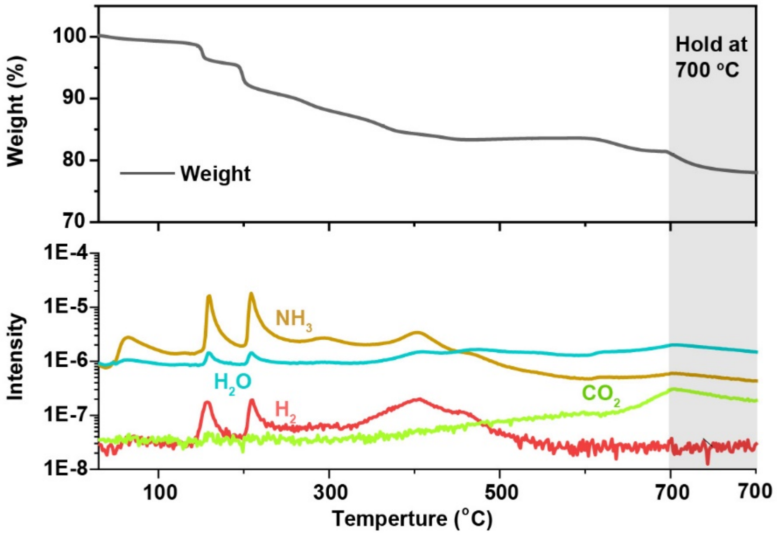

Supplementary Fig. 7. TG-MS results of AMBN/$NaNH_2$ mixture from room temperature to 700 °C. Source data are provided as a Source Data file.

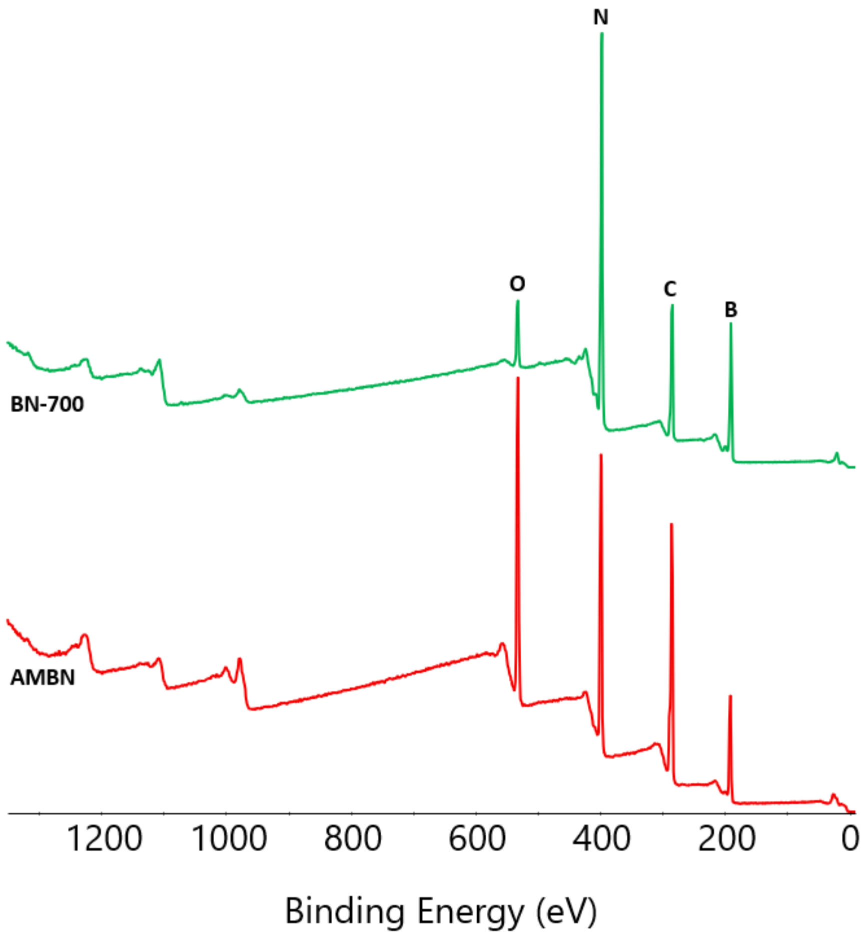

Supplementary Fig. 8. XPS survey spectra of AMBN and BN-700. Source data are provided as a Source Data file.

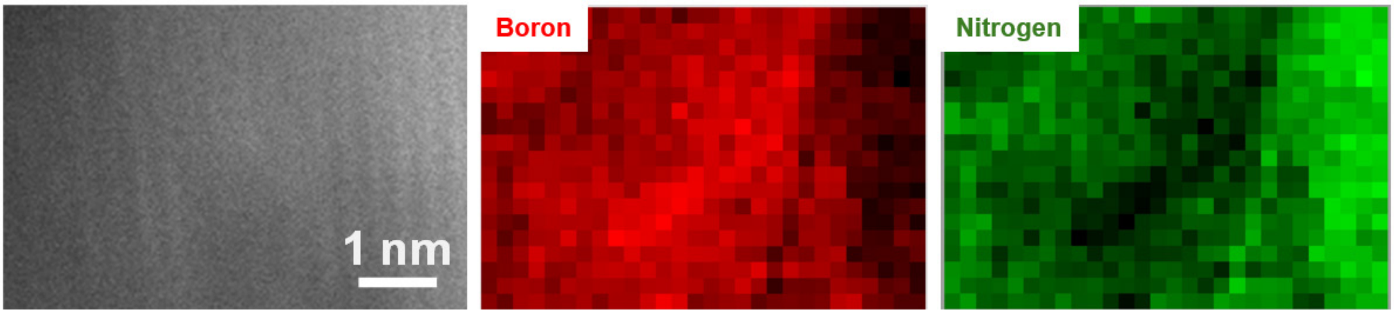

Supplementary Fig. 9. HAADF STEM image of BN-700, and corresponding B and N element distribution from EELS.

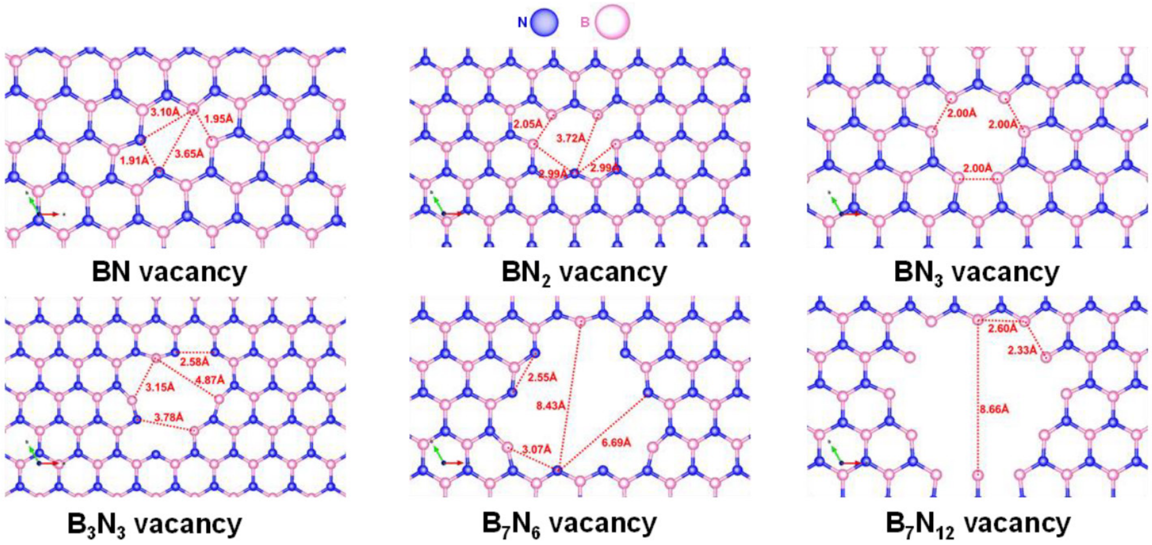

Supplementary Fig. 10. BN models with different defect structures based on theoretical study from previous work.[S1] Boron, and nitrogen atoms are represented by pink, and blue spheres, respectively. Reprinted with permission from Ref. S1. Copyright 2022 American Chemical Society.

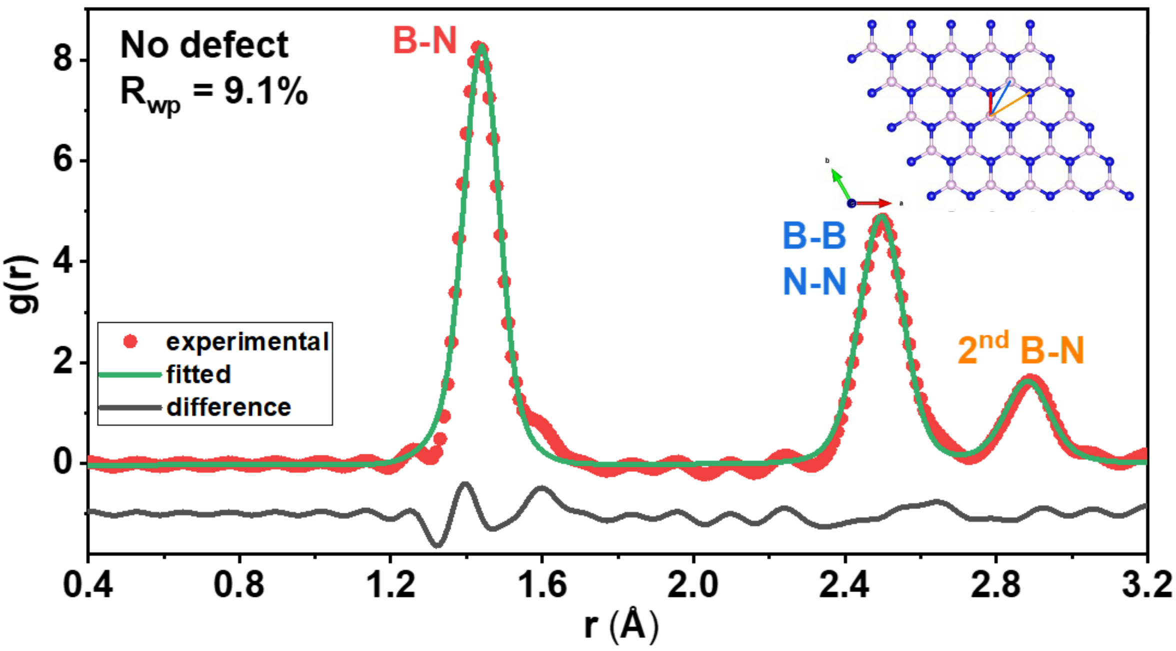

Supplementary Fig. 11. Comparison of the neutron PDF results of BN model without defect structure with BN-700. Boron, and nitrogen atoms are represented by pink, and blue spheres, respectively. Source data are provided as a Source Data file.

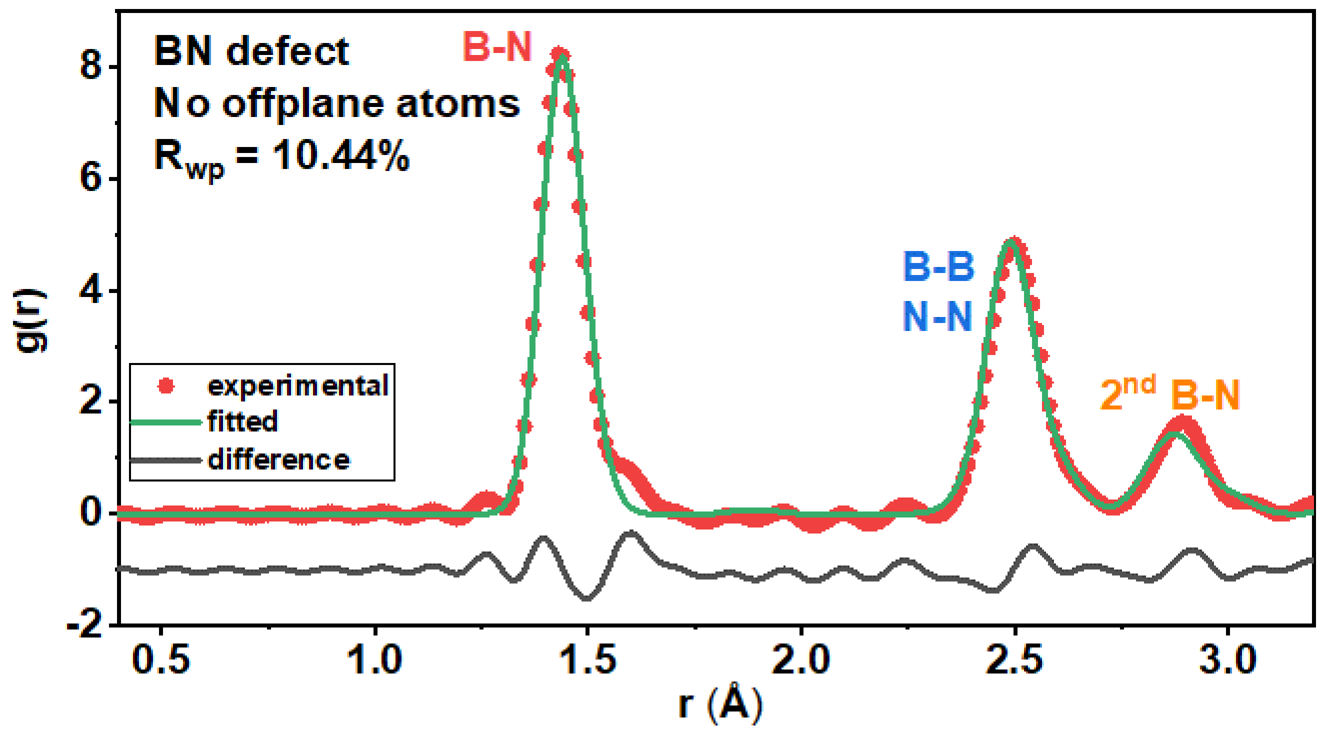

Supplementary Fig. 12. Comparison of the neutron PDF results of BN model with BN defect structure with BN-700. No offplane atoms were involved. Source data are provided as a Source Data file.

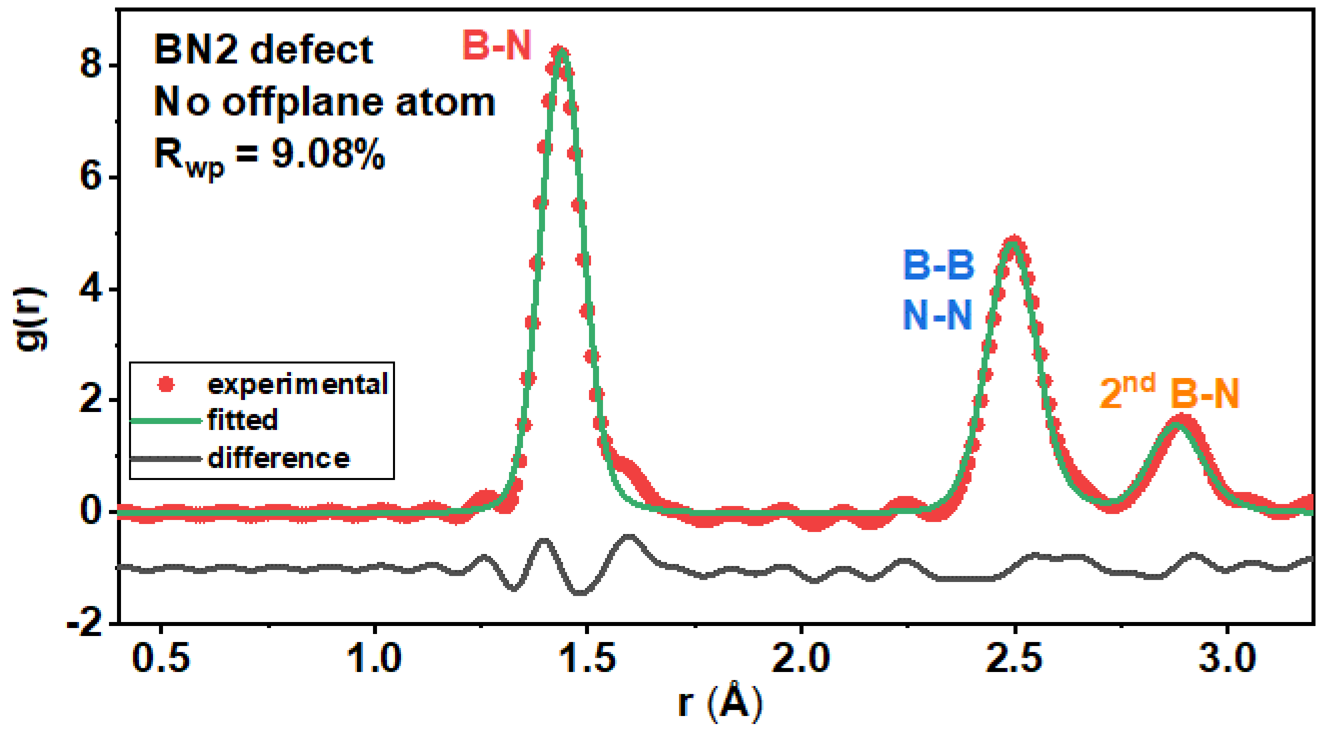

Supplementary Fig. 13. Comparison of the neutron PDF results of BN model with BN2 defect structure with BN-700. No offplane atoms were involved. Source data are provided as a Source Data file.

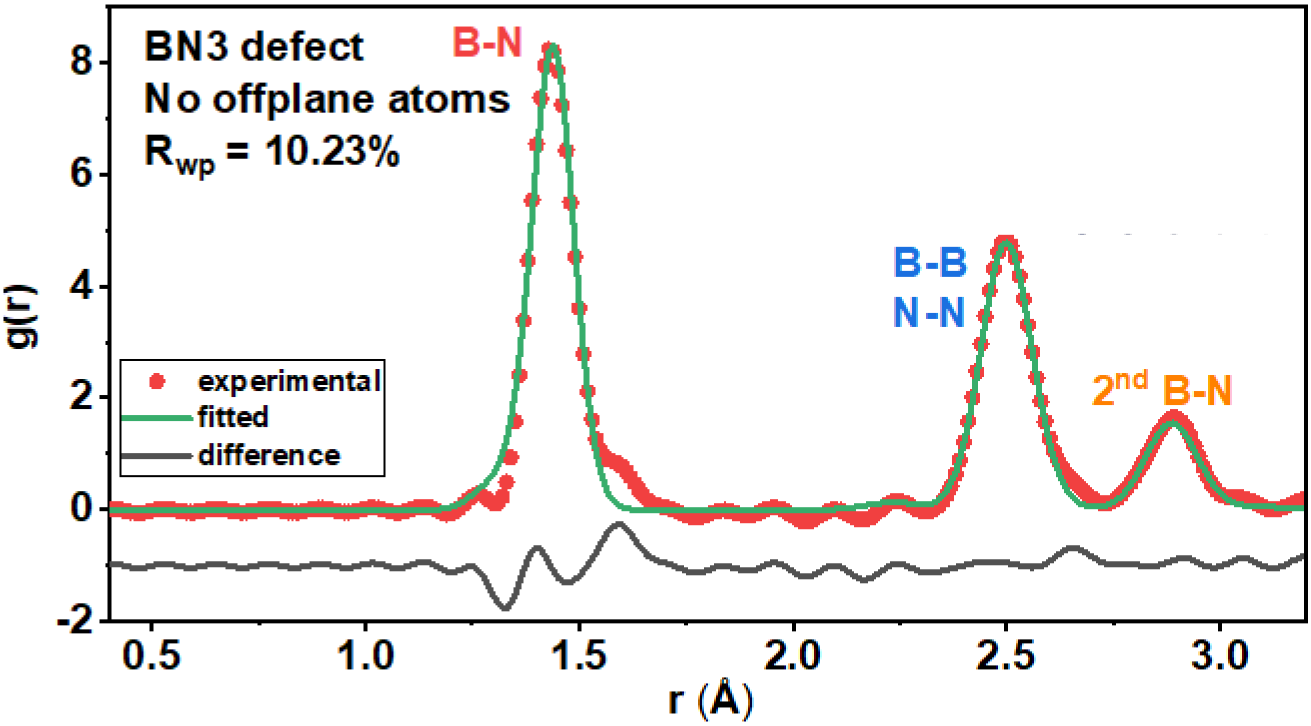

Supplementary Fig. 14. Comparison of the neutron PDF results of BN model with BN3 defect structure with BN-700. No offplane atoms were involved. Source data are provided as a Source Data file.

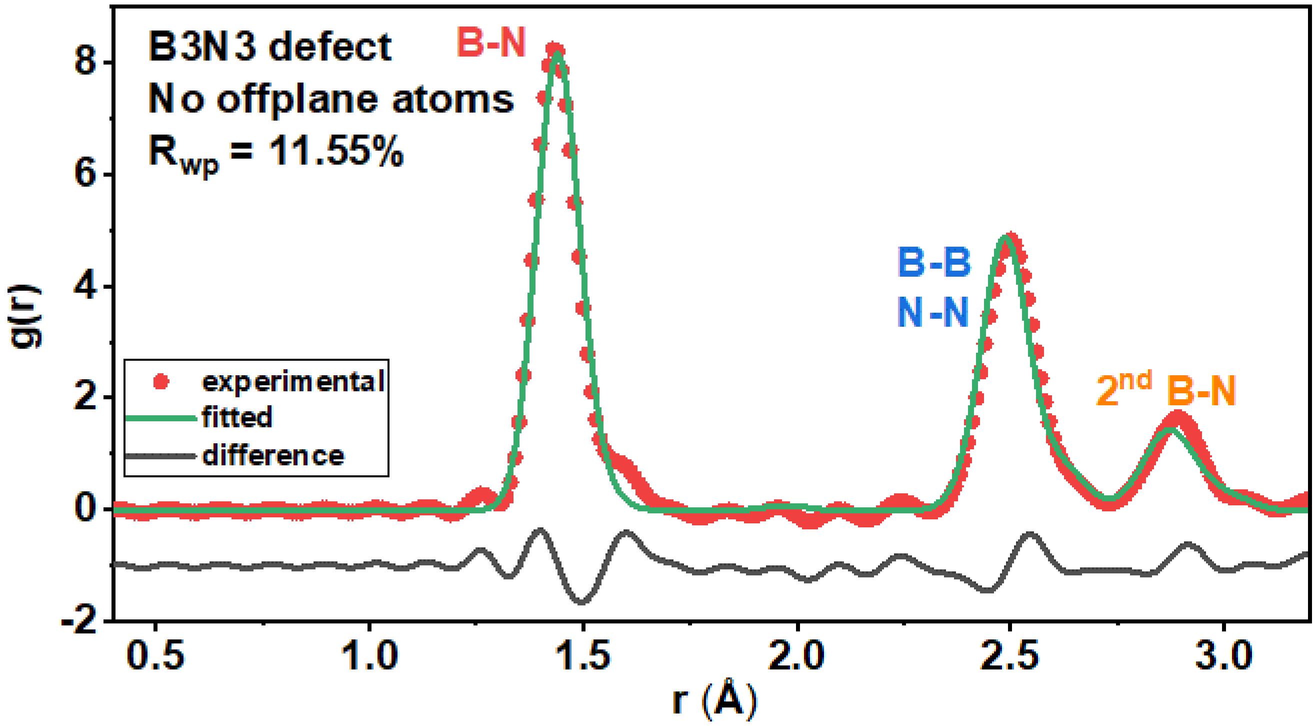

Supplementary Fig. 15. Comparison of the neutron PDF results of B3N3 model with BN3 defect structure with BN-700. No offplane atoms were involved. Source data are provided as a Source Data file.

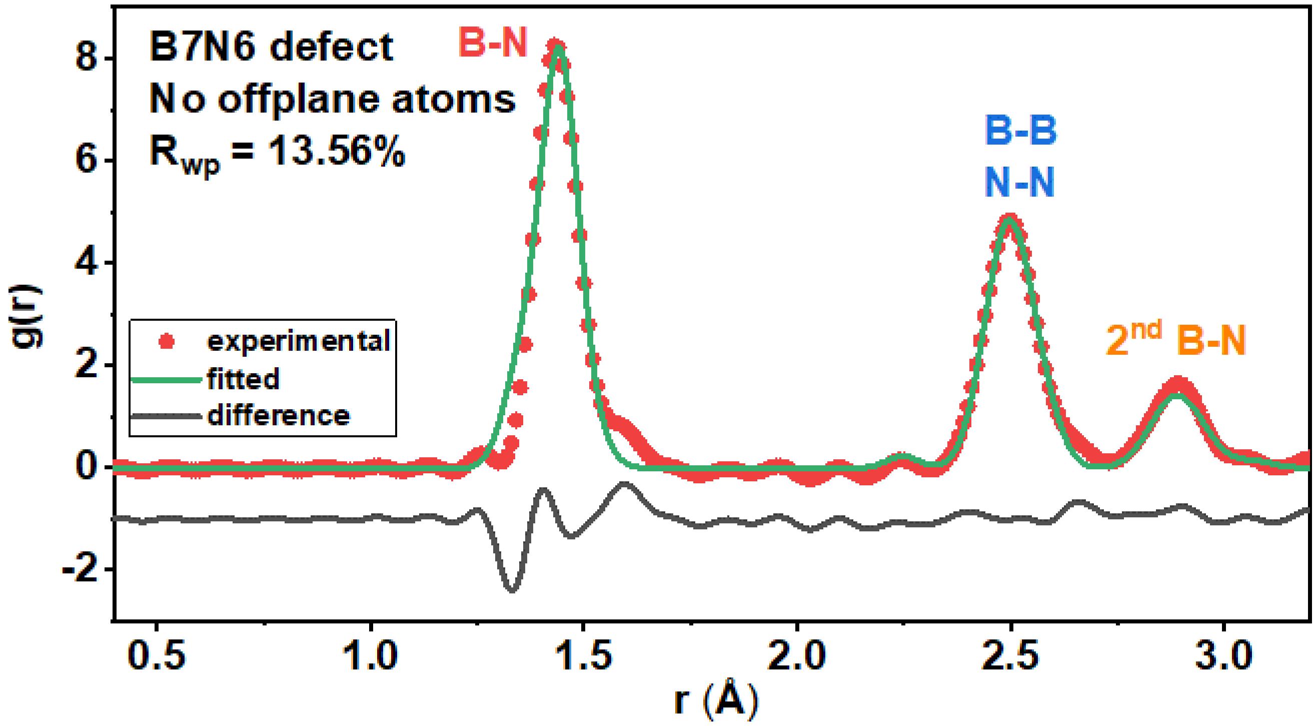

Supplementary Fig. 16. Comparison of the neutron PDF results of B7N6 model with BN3 defect structure with BN-700. No offplane atoms were involved. Source data are provided as a Source Data file.

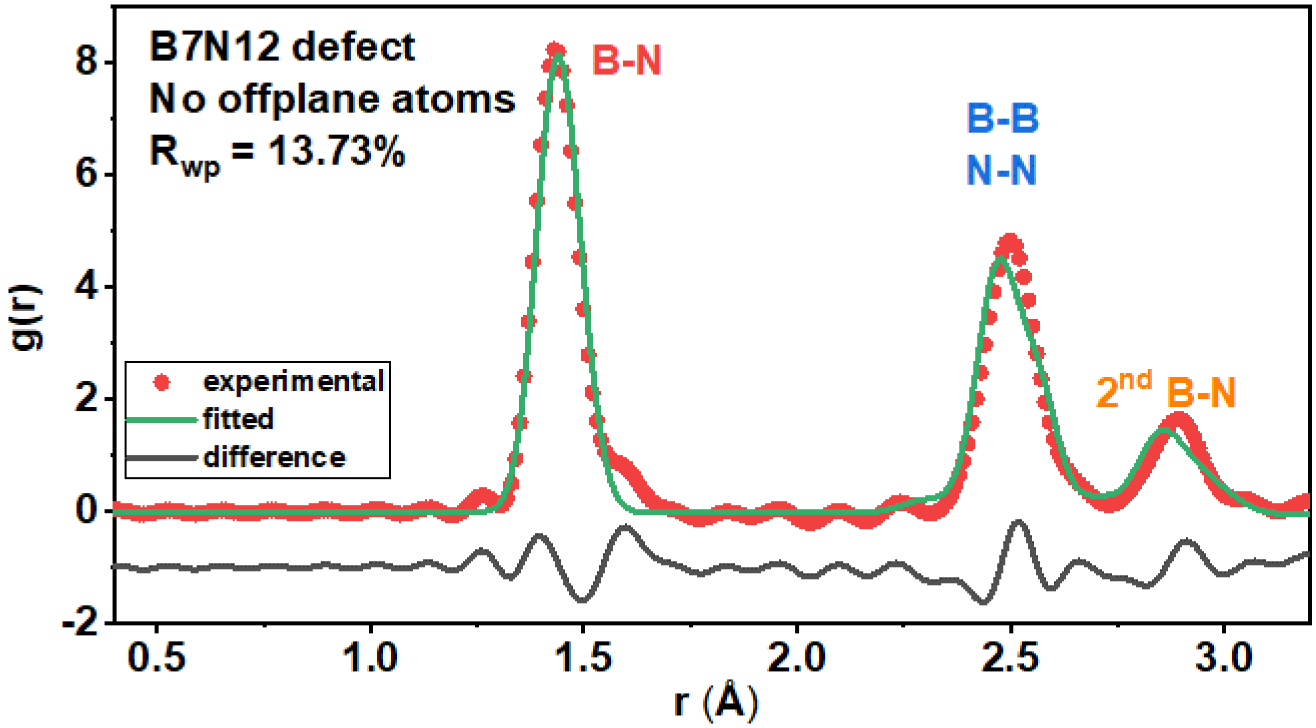

Supplementary Fig. 17. Comparison of the neutron PDF results of B7N12 model with BN3 defect structure with BN700. No off-plane atoms were involved. Source data are provided as a Source Data file.

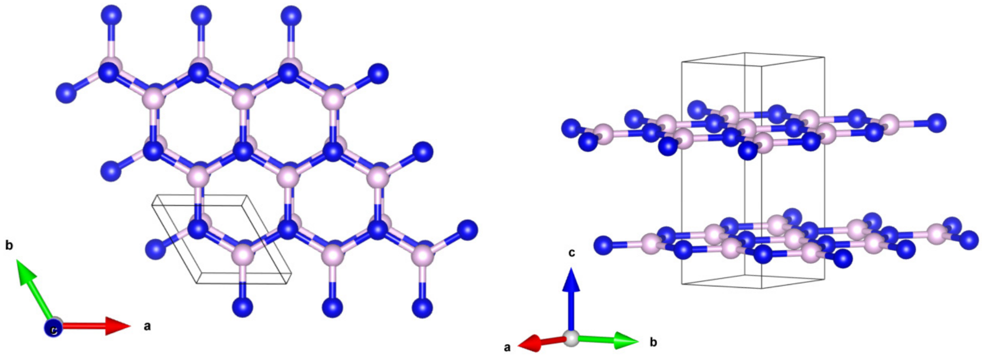

Supplementary Fig. 18. Literature reports of the stacking of the B-N layers along c-axis. The most broadly cited model is the bi-layer A-a stacking model (B and N facing each other on the adjacent layers) with the space group of $P6_3$/mmc. Boron, and nitrogen atoms are represented by pink, and blue spheres, respectively.

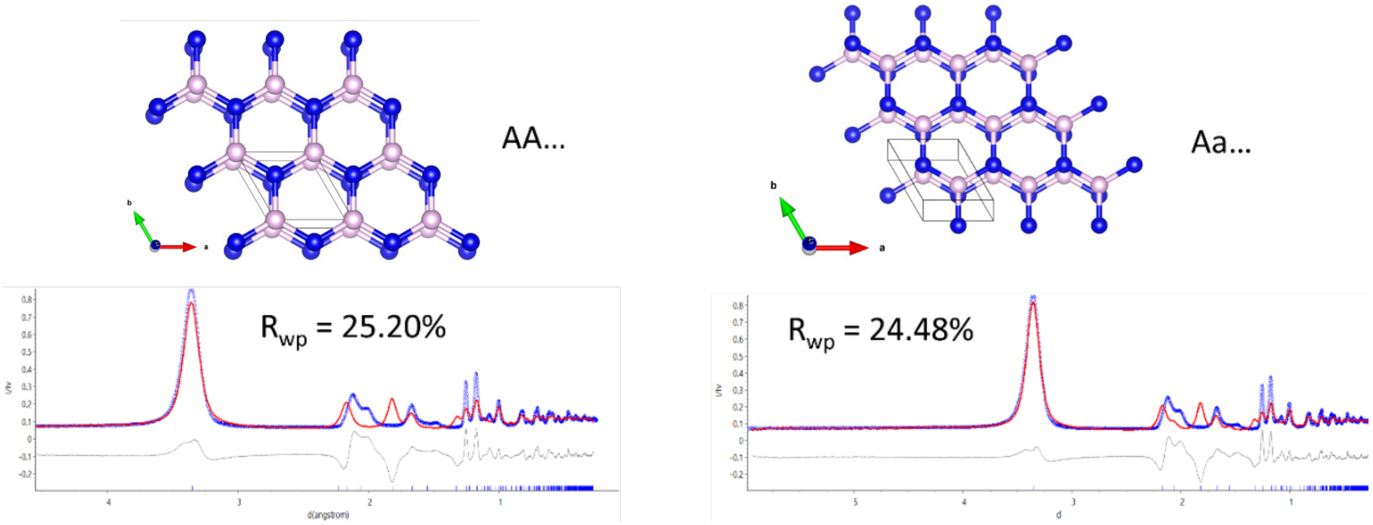

Supplementary Fig. 19. Structure refinements using the AA (boron facing boron, and N facing N in the successive layers) or Aa (B-N alternative stacking in the successive layers) stacking of B-N layers. The fits are very poor, with the residual R_{wp} larger than 20%. Neutron diffraction data were collected at NOMAD. The plots here shown the data from the 2θ = 32° bank. Boron, and nitrogen atoms are represented by pink, and blue spheres, respectively. Source data are provided as a Source Data file.

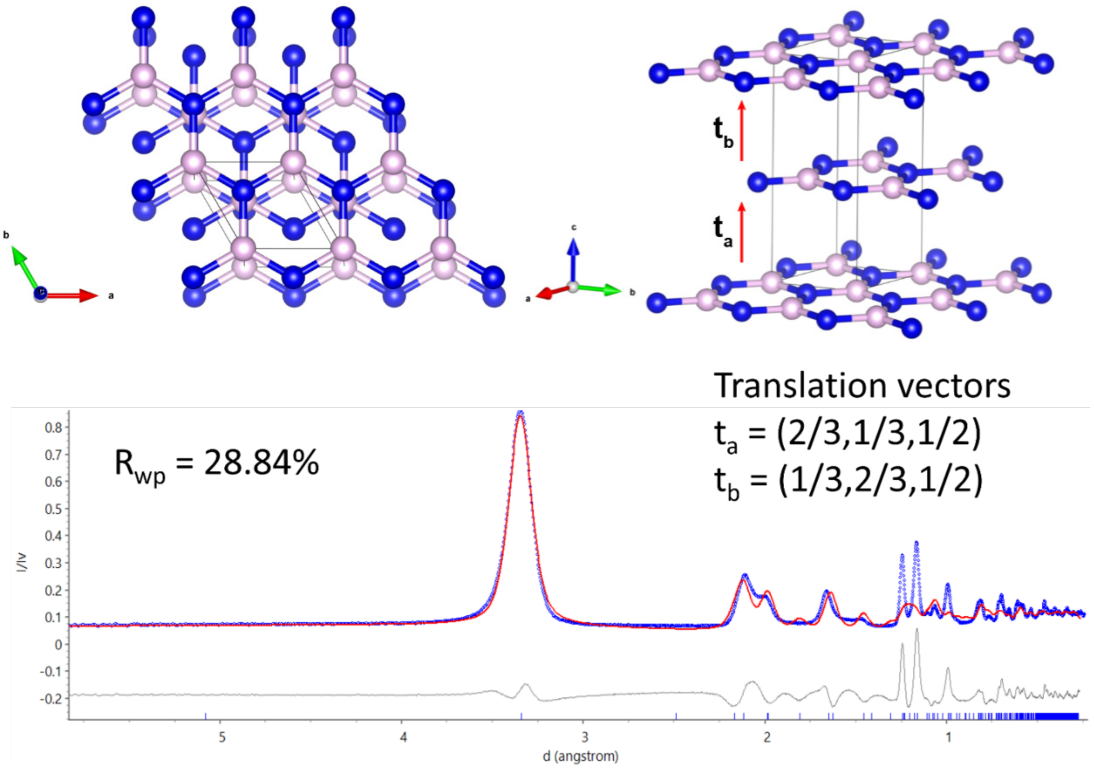

Supplementary Fig. 20. Structure refinements using the AB stacking of B-N layers. The fit is even worse than the Aa or AA stacking. Boron, and nitrogen atoms are represented by pink, and blue spheres, respectively. Source data are provided as a Source Data file.

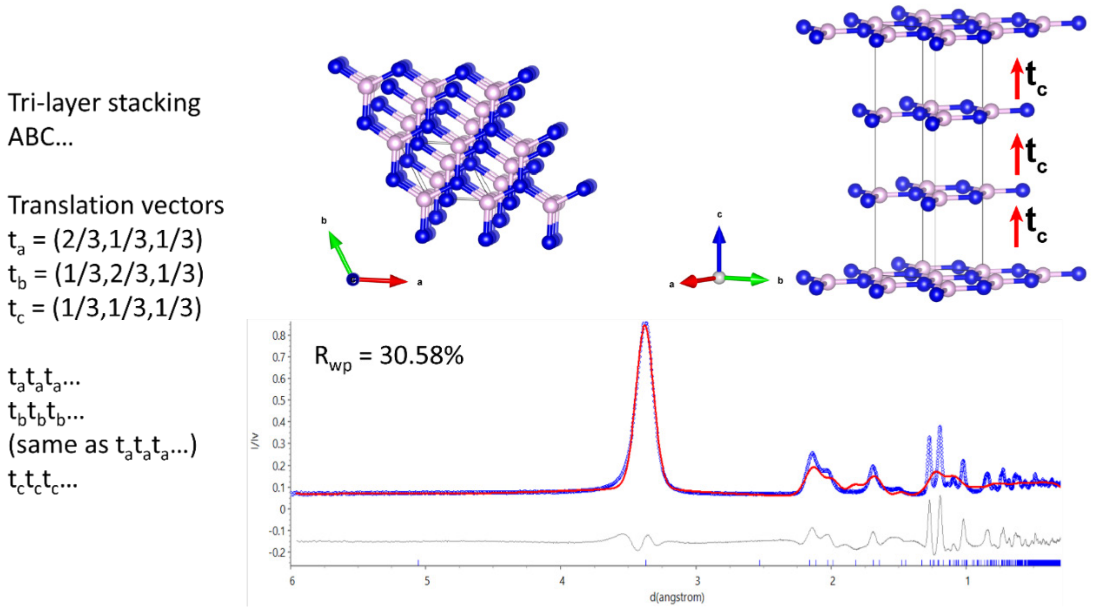

Supplementary Fig. 21. Structure refinements using the ABC… tri-layer stacking of B-N layers. There are three plausible translation vectors for the ABC… type stacking. $t_at_at_a$ and $t_bt_bt_b$ type stacking leads to the same structure while $t_ct_ct_c$… results in a different stacked structure. The above figures shows that pure $t_ct_ct_c$… type stacking results in poor quality fit. Boron, and nitrogen atoms are represented by pink, and blue spheres, respectively. Source data are provided

as a Source Data file.

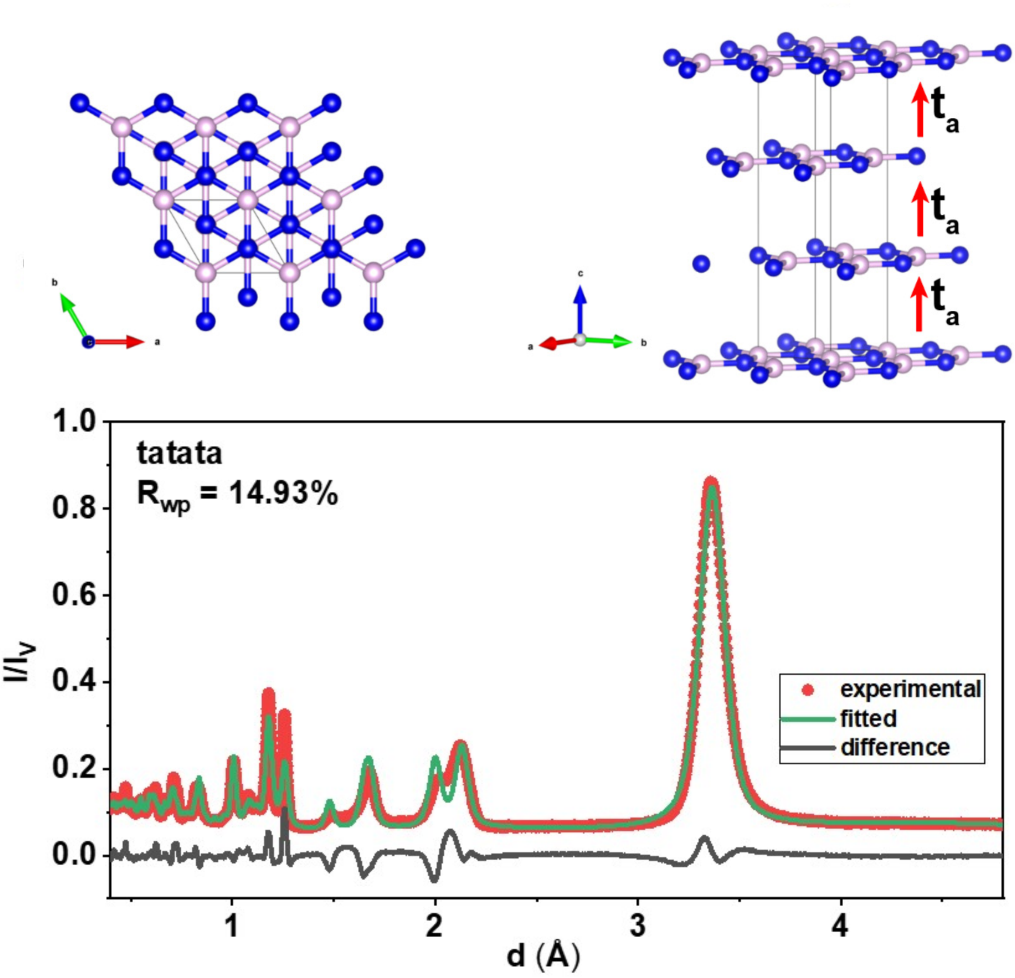

Supplementary Fig. 22. Structure refinements of BN-700 using the $t_at_at_a$… type stacking. Much better fit can be achieved with this type of tri-layer stacking. However, there is still noticeable discrepancies in the fit, very likely due to the presence of stacking faults. Boron, and nitrogen atoms are represented by pink, and blue spheres, respectively. Source data are provided as a Source Data file.

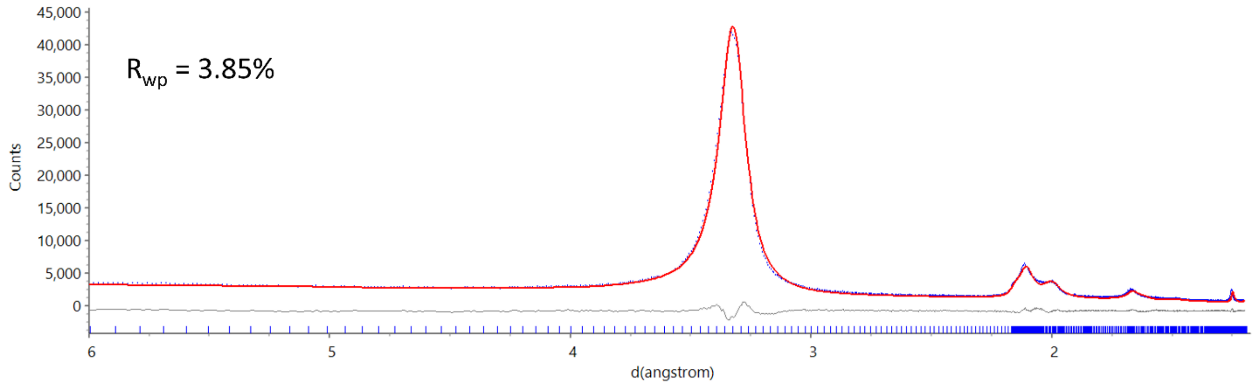

Supplementary Fig. 23. Comparison of the PXRD patterns of BN model with off-plane BN2 defects and BN-700. Source data are provided as a Source Data file.

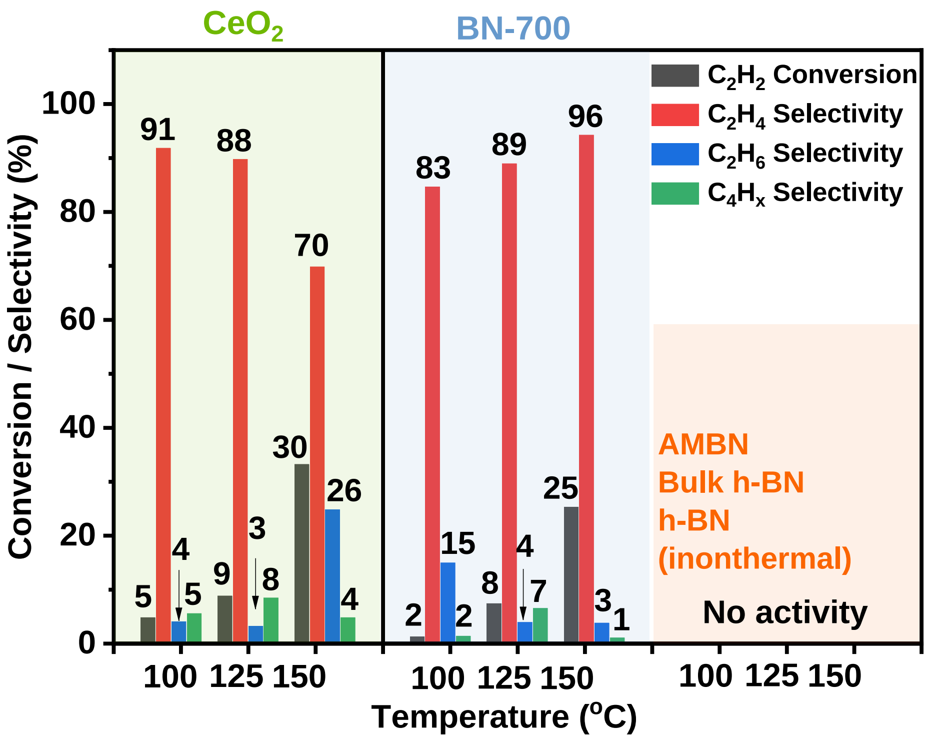

Supplementary Fig. 24. Comparison the activity of BN-700, CeO_2, and other BNs (AMBN, commercial bulk h-BN, and h-BN nanoparticles synthesized through ionothermal method) in acetylene hydrognation at 100, 125, and 150 °C. Source data are provided as a Source Data file.

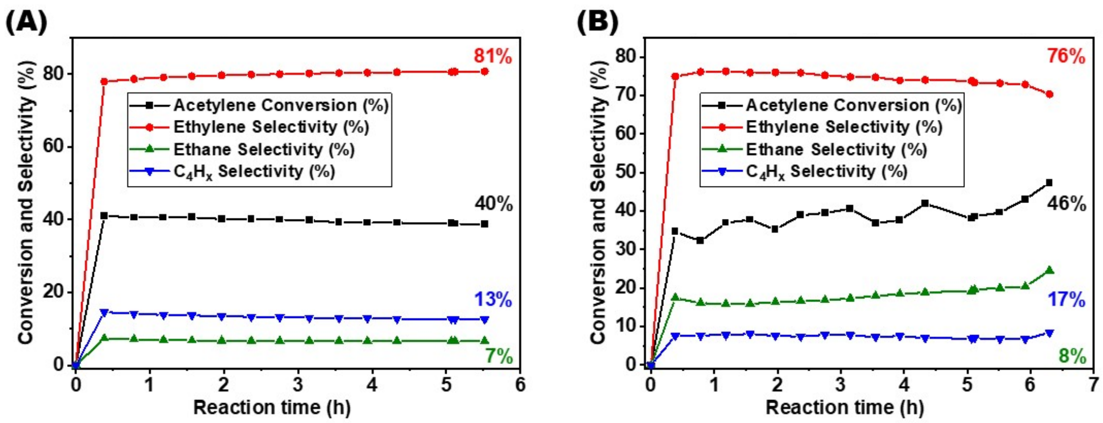

Supplementary Fig. 25. Screening of reaction parameters for acetylene hydrogenation catalyzed by BN-700. (A) 75 mg of BN-700 catalyst using a 25 ccm flow of 0.5% C_2H_2 / 16% H_2 / 83.5% Ar at 150 °C. (B) 300 mg of BN-700 catalyst using a 50 ccm flow of 0.5% C_2H_2 / 16% H_2 / 83.5% Ar at 150 °C. Source data are provided as a Source Data file.

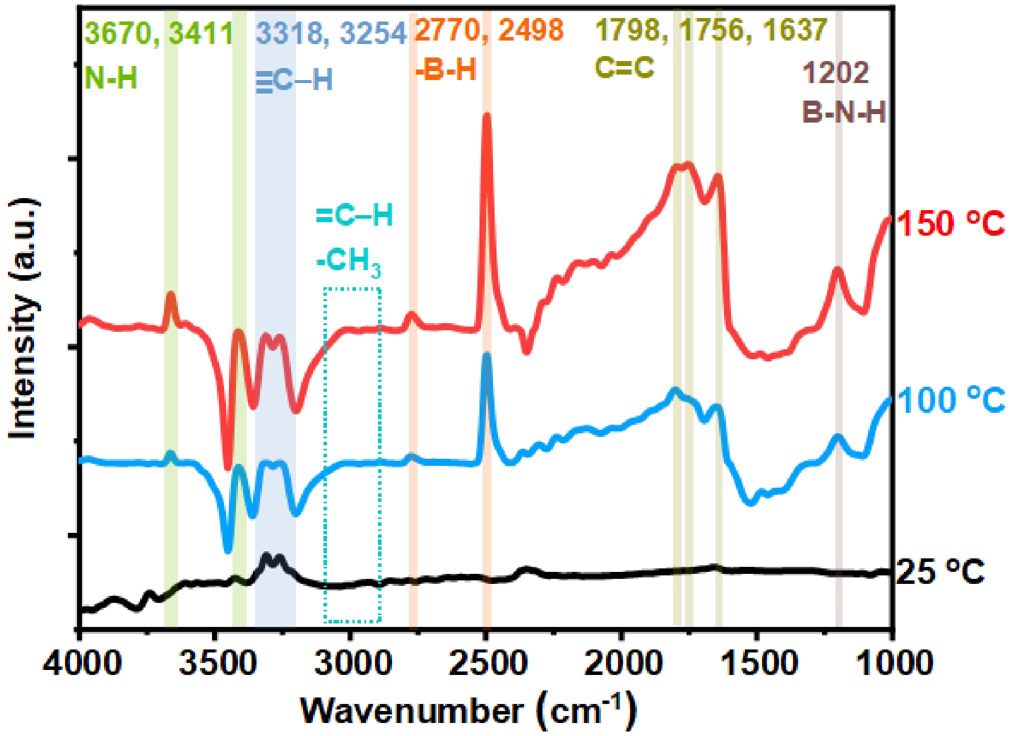

Supplementary Fig. 26. In-situ acetylene semi-hydrogenation DRIFTS desorption spectra on BN-700 catalyst at 25, 100, and 150 ºC. Source data are provided as a Source Data file.

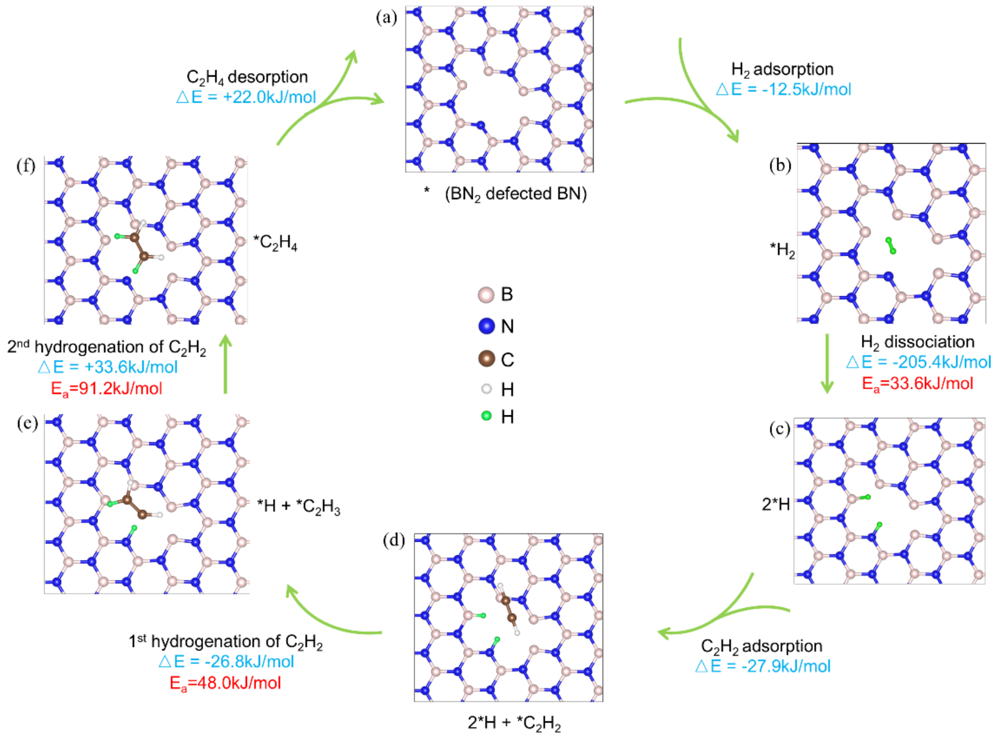

Supplementary Fig. 27. Proposed acetylene semi-hydrogenation mechanism based on BN model with BN_2 defect. Hydrogen, carbon, boron, and nitrogen atoms are represented by white, brown, pink, and blue spheres, respectively. Hydrogen atoms part in the reaction were highlighted as green spheres.

Supplementary Tables

Supplementary Table 1 Summary of H_2 adsorption energies on various boron nitride materials based on theoretical simulations.

Sample	Defect types	Absorbate	Energy type	Value from simulation	Reference
(10,0) boron nitride Nanotubes	B sites and N sites (no defects)	Hydrogen molecule binding energies	Adsorption energy	-0.085 and -0.1 eV	S2
(9,9) armchair-type boron nitride nanotubes	B sites and N sites (no defects)	Hydrogen molecule binding energies	Adsorption energy	-0.027 and -0.037 eV	S3
boron nitride nanotubes	V_N, V_B	Hydrogen atoms	Adsorption energy	-5.24 and -9.40 eV	S4
hexagonal boron nitride	V_N, V_B	Hydrogen atoms	Adsorption energy	-1.55 and -5.18 eV	S5
hexagonal boron nitride[a]	BN, BN2, BN3, B3N3, B7N6, B7N12	Hydrogen atoms	Dissociative H_2 adsorption energy	-5.1 to 0.5 eV	S6
hexagonal boron nitride[b]	2V(1B1N), 3V(2B1N), 3V(1B2N), 4V (3B1N), 6V(3B3N)	Hydrogen atoms	Gibbs free energy of hydrogenation	-4.0 to -0.5 eV	S7
Carbon-doped hexagonal boron nitride[c]	C_B(N,N,N), C_B(C,N,N), C_B(C,C,N), C_B(C,C,C), C_N(B,B,B), C_N(C,B,B), C_N(C,C,B), C_N(C,C,C)	Hydrogen molecule	Adsorption energy	-0.084 to -0.063 eV	S8
Li^+ doped boron nitride nanotubes	NA	Hydrogen molecule	Adsorption energy	-0.33 eV	S9
lithium decorated 3D hybrid Boron-Nitride-Carbon frameworks	NA	Hydrogen molecule	Adsorption energy	-0.24 eV	S10
Ti-doped boron nitride	NA	Hydrogen molecule	Adsorption energy	-0.70 eV	S11

[a] Structure of boron nitride with defects shown in Supplementary Fig. 10.
[b] Structure of boron nitride with defects.

	[c] Structure of carbon-doped boron nitride.

Supplementary Table 2 Selected examples of the previously reported heterogenous and homogeneous catalytic FLP systems.

FLP catalyst	Acid sites	Base sites	Reaction	Ref.
Heterogeneous	B sites	N sites	Hydrogenation of alkenes and alkynes	S12
Heterogeneous	B sites	N sites	Hydrogenation of alkenes and alkynes	S13
Heterogeneous	B sites	N sites	Olefin hydrogenation via mechanochemistry	S14
Heterogeneous	B sites	N sites	Selective Hydrogenation	S15
Heterogeneous	B sites	N sites	Electrocatalytic nitrogen reduction	S16
Heterogeneous	C sites	N sites	Dehydrogenation	S17
Heterogeneous	C cites	Heteroatoms	Alkene hydrogenation	S18
Heterogeneous	surface defects (adjacent surface Ce^{3+})	surface lattice oxygen	Hydrogenation of styrene	S19
Heterogeneous	In(III) sites	InOH sites	Hydrogenation of CO2	S20
Semi-heterogeneous	$B(C_6F_5)_3$	Oxygen atoms on the surface of the α-cyclodextrin	Hydrogenation, Deoxygenation	S21
Semi-heterogeneous	$B(C_6F_5)_3$	Pyridyl sites	Hydrogenation of ketones	S22
Homogeneous	$B(C_6F_5)_3$	electron-rich B atoms	Hydrogenation	S23
Homogeneous	2,2,6,6-tetramethylpiperidine (TMP)	B in H- $B(C_6F_5)_3$	Hydrogenation of CO_2 to CH_3OH	S24

Supplementary Table 3. Assignment of Vibrational Bands in the DRIFTS Spectra of C_2H_2 Adsorption and Reaction on BN-700 surface.

Surface Adsorbate	Assignment	Band (cm^{-1})	Reference
Weakly absorbed C_2H_2 via H-bond	v_{as}(N-H)	3670	S25, S26
Acetylene	v(≡C－H)	3300-3200	S25, S27
Acetylide (−C≡C–H)	v_{as}(C–H)	3250	S27, S28
Surface Boron Hydride	v(B-H_{ads})	2500	S26, S28
Protonated Acetylene $H^+(C_2H_2)$	v_{as}(C≡C)	2200	S29
π-bonded CH_2═CH_2	v(C═C)	1700-1660	S25, S30 , S31

Supplementary Table 4. Comparison of H_2 activation energy on different noble-metal free catalysts.

Catalyst	Material	H_2 activation energy (kJ/mol)	Reference
**BN-700**	BN	58.2	This work
**CeO_2**	CeO_2	70.7	This work
**CeO_2**	CeO_2	96	S32
**$BH_2CH_2NH_2$**	model molecule	50.2	S33
**BH_2NH_2**	model molecule	178.6	S33
**BH_2PH_2**	model molecule	77.4	S33
**T-H600**	TiO_2	56.4	S34
**TiO_2-fresh catalyst**	TiO_2	72	S34
**Cu_1/PHI**	Cu on poly(heptazine imide)	70.9	S35

Reference

S1 Chen, H. *et al.* Defect-Regulated Frustrated-Lewis-Pair Behavior of Boron Nitride in Ambient Pressure Hydrogen Activation. *J. Am. Chem. Soc.* **144**, 10688-10693 (2022). https://doi.org/10.1021/jacs.2c00343

S2 Jhi, S.-H. & Kwon, Y.-K. Hydrogen adsorption on boron nitride nanotubes: A path to room-temperature hydrogen storage. *Phys. Rev. B* **69** (2004). https://doi.org/10.1103/PhysRevB.69.245407

S3 Mpourmpakis, G. & Froudakis, G. E. Why boron nitride nanotubes are preferable to carbon nanotubes for hydrogen storage?: An ab initio theoretical study. *Catalysis Today* **120**, 341-345 (2007). https://doi.org/10.1016/j.cattod.2006.09.023

S4 Wu, X., Yang, J., Hou, J. G. & Zhu, Q. Defects-enhanced dissociation of H2 on boron nitride nanotubes. *J. Chem. Phys.* **124**, 054706 (2006). https://doi.org/10.1063/1.2162897

S5 Shevlin, S. A. & Guo, Z. X. Hydrogen sorption in defective hexagonal BN sheets and BN nanotubes. *Phys. Rev. B* **76** (2007). https://doi.org/10.1103/PhysRevB.76.024104

S6 Chen, H. *et al.* Defect-Regulated Frustrated-Lewis SI. *J. Am. Chem. Soc.* **144**, 10688-10693 (2022). https://doi.org/10.1021/jacs.2c00343

S7 Sassi, M. & Autrey, T. First-Principles Study of Molecular Hydrogen Activation by Defects in Boron Nitride. *J. Phys. Chem. C* **129**, 6657-6665 (2025). https://doi.org/10.1021/acs.jpcc.5c00806

S8 Ghosh, S., Nath, P., Moshat, S. & Sanyal, D. Role of carbon substitutional and vacancy in tailoring the H2 adsorption energy over a hexagonal boron nitride monolayer: an ab initio study. *Journal of Materials Science* **59**, 10877-10887 (2024). https://doi.org/10.1007/s10853-024-09807-x

S9 Panigrahi, P. *et al.* Capacity enhancement of polylithiated functionalized boron nitride nanotubes: an efficient hydrogen storage medium. *Phys Chem Chem Phys* **22**, 15675-15682 (2020). https://doi.org/10.1039/d0cp01237h

S10 Bi, L., Yin, J., Huang, X., Wang, Y. & Yang, Z. A DFT study of H2 adsorption on lithium decorated 3D hybrid Boron-Nitride-Carbon frameworks. *International Journal of Hydrogen Energy* **44**, 15183-15192 (2019). https://doi.org/10.1016/j.ijhydene.2019.04.114

S11 Shevlin, S. A. & Guo, Z. X. Transition-metal-doping-enhanced hydrogen storage in boron nitride systems. *Applied Physics Letters* **89** (2006). https://doi.org/10.1063/1.2360232

S12 Li, M. *et al.* Construction of Boron- and Nitrogen-Enriched Nanoporous pi-Conjugated Networks Towards Enhanced Hydrogen Activation. *Angew. Chem. Int. Ed.* **62**, e202302684 (2023). https://doi.org/10.1002/anie.202302684

S13 Chen, H. *et al.* Defect-Regulated Frustrated-Lewis-Pair Behavior of Boron Nitride in Ambient Pressure Hydrogen Activation. *Journal of the American Chemical Society* **144**, 10688-10693 (2022). https://doi.org/10.1021/jacs.2c00343

S14 Nash, D. J. *et al.* Heterogeneous Metal-Free Hydrogenation over Defect-Laden Hexagonal Boron Nitride. *ACS Omega* **1**, 1343-1354 (2016). https://doi.org/10.1021/acsomega.6b00315

S15 Ding, Y. *et al.* A Heterogeneous Metal-Free Catalyst for Hydrogenation: Lewis Acid–Base Pairs Integrated into a Carbon Lattice. *Angew. Chem. Int. Ed.* **57**, 13800-13804 (2018). https://doi.org/https://doi.org/10.1002/anie.201803977

S16 Lin, W. *et al.* Creating Frustrated Lewis Pairs in Defective Boron Carbon Nitride for Electrocatalytic Nitrogen Reduction to Ammonia. *Angew. Chem. Int. Ed.* **61**, e202207807 (2022). https://doi.org/https://doi.org/10.1002/anie.202207807

S17 Hu, H. *et al.* Metal-free carbocatalyst for room temperature acceptorless dehydrogenation of N-heterocycles. *Sci. Adv.* **8**, eabl9478 (2022). https://doi.org/10.1126/sciadv.abl9478

S18 Primo, A., Neatu, F., Florea, M., Parvulescu, V. & Garcia, H. Graphenes in the absence of metals as carbocatalysts for selective acetylene hydrogenation and alkene hydrogenation. *Nat. Commun.* **5**, 5291 (2014). https://doi.org/10.1038/ncomms6291

S19 Zhang, S. *et al.* Solid frustrated-Lewis-pair catalysts constructed by regulations on surface defects of porous nanorods of CeO2. *Nat. Commun.* **8**, 15266 (2017). https://doi.org/10.1038/ncomms15266

S20 Ghuman, K. K. *et al.* Surface Analogues of Molecular Frustrated Lewis Pairs in Heterogeneous CO2 Hydrogenation Catalysis. *ACS Catal.* **6**, 5764-5770 (2016). https://doi.org/10.1021/acscatal.6b01015

S21 Mahdi, T. & Stephan, D. W. Facile Protocol for Catalytic Frustrated Lewis Pair Hydrogenation and Reductive Deoxygenation of Ketones and Aldehydes. *Angew. Chem. Int. Ed.* **54**, 8511-8514 (2015). https://doi.org/https://doi.org/10.1002/anie.201503087

S22 Tian, C. *et al.* Use of steric encumbrance to develop conjugated nanoporous polymers for metal-free catalytic hydrogenation. *Chem. Commun.* **52**, 11919-11922 (2016). https://doi.org/10.1039/C6CC06372A

S23 Welch, G. C., Juan, R. R. S., Masuda, J. D. & Stephan, D. W. Reversible, Metal-Free Hydrogen Activation. *Science* **314**, 1124-1126 (2006). https://doi.org/10.1126/science.1134230

S24 Ashley, A. E., Thompson, A. L. & O'Hare, D. Non-Metal-Mediated Homogeneous Hydrogenation of CO2 to CH3OH. *Angew. Chem. Int. Ed.* **48**, 9839-9843 (2009). https://doi.org/10.1002/anie.200905466

S25 Moon, J. *et al.* Discriminating the role of surface hydride and hydroxyl for acetylene semihydrogenation over ceria through in situ neutron and infrared spectroscopy. *ACS Catal.* **10**, 5278-5287 (2020).

S26 Chen, H. *et al.* Defect-regulated Frustrated-Lewis-Pair behavior of boron nitride in ambient pressure hydrogen activation. *J. Am. Chem. Soc.* **144**, 10688-10693 (2022).

S27 Ivanov, A. V., Koklin, A. E., Uvarova, E. B. & Kustov, L. M. A DRIFT spectroscopic study of acetylene adsorbed on metal oxides. *Physical Chemistry Chemical Physics* **5**, 4718-4723 (2003).

S28 Tominaka, S. *et al.* Geometrical frustration of BH bonds in layered hydrogen borides accessible by soft chemistry. *Chem* **6**, 406-418 (2020).

S29 Douberly, G. E. *et al.* Infrared photodissociation spectroscopy of protonated acetylene and its clusters. *J. Phys. Chem. A* **112**, 1897-1906 (2008).

S30 Cao, T. *et al.* An in situ DRIFTS mechanistic study of CeO 2-catalyzed acetylene semihydrogenation reaction. *Physical Chemistry Chemical Physics* **20**, 9659-9670 (2018).

S31 Carrasco, J. *et al.* Molecular-level understanding of CeO2 as a catalyst for partial alkyne hydrogenation. *J. Phys. Chem. C* **118**, 5352-5360 (2014).

S32 Vilé, G. *et al.* Promoted ceria catalysts for alkyne semi-hydrogenation. *J. Catal.* **324**, 69-78 (2015).

S33 Wang, Z., Lu, G., Li, H. & Zhao, L. Encumbering the intramolecular π donation by using a bridge: A strategy for designing metal-free compounds to hydrogen activation. *Chin. Sci. Bull.* **55**, 239-245 (2010).

S34 Wan, Q., Chen, Y., Zhou, S., Lin, J. & Lin, S. Selective hydrogenation of acetylene to ethylene on anatase TiO2 through first-principles studies. *J. Mater. Chem. A* **9**, 14064-14073 (2021).

S35 Chen, X., Li, Y., Yuan, Y. & Lin, W. What size of Cun clusters loaded on poly(heptazine imide) have better catalytic performance for acetylene semi-hydrogenation? *Mol. Catal.* **569**, 114605 (2024). https://doi.org/https://doi.org/10.1016/j.mcat.2024.114605
