# Supplementary material for: Selective semihydrogenation of acetylene in ethylene using defect-rich boron nitride catalyst from flux reconstruction
Source: Nat Commun. 2025 Nov 12;16:9948. doi: 10.1038/s41467-025-64886-x (PMC12612240; doi:10.1038/s41467-025-64886-x)
Supplement: Supplementary file 2 — Supplementary Data 1 [file 41467_2025_64886_MOESM2_ESM.zip › Legend for supplementary data 1.docx]

Structure used for DFT calculation is provided in the text file.
